# Supplementary material for: Perceptions of institutional performance and compliance to non-pharmaceutical interventions: How performance perceptions and policy compliance affect public health in a decentralized health system
Source: PLoS One. 2023 May 12;18(5):e0285289. doi: 10.1371/journal.pone.0285289 (PMC10180683; doi:10.1371/journal.pone.0285289)
Supplement: S1 Table — (DOCX) [file pone.0285289.s001.docx]

**S1 Table. Survey Questions**

| **Questions** | | | | | | | | | | | | | | | | | | | **%** |
| --- | --- | --- | --- | --- | --- | --- | --- | --- | --- | --- | --- | --- | --- | --- | --- | --- | --- | --- | --- |
| **What is your perception about the performance of the following institutions:** | | | | | | | | | | | | | | | | | | |  |
|  | | | *Very Bad* | | | | | *Bad* | | *Average* | | | *Good* | | | *Very Good* | | *No answer* | |
| Federal Government | | | 41.7 | | | | | 19.7 | | 21.6 | | | 10.6 | | | 3.9 | | 2.5 | |
| State Government | | | 16.7 | | | | | 20.1 | | 33.7 | | | 20.6 | | | 5.4 | | 3.5 | |
| Municipal Government | | | 16.2 | | | | | 20.0 | | 34.7 | | | 19.9 | | | 5.1 | | 4.2 | |
| National Health System | | | 4.2 | | | | | 6.4 | | 23.7 | | | 35.3 | | | 25.4 | | 5.1 | |
| Health Ministry | | | 23.8 | | | | | 22.2 | | 28.6 | | | 17.8 | | | 4.9 | | 2.8 | |
| Federal Court | | | 20.8 | | | | | 24.5 | | 32.0 | | | 10.5 | | | 1.2 | | 11.0 | |
| National Congress | | | 25.0 | | | | | 29.9 | | 29.5 | | | 6.3 | | | 0.6 | | 8.8 | |
| World Health Organization | | | 7.0 | | | | | 8.2 | | 26.8 | | | 36.3 | | | 17.8 | | 3.8 | |
| Formal Media | | | 12.3 | | | | | 11.9 | | 26.6 | | | 31.9 | | | 14.9 | | 2.4 | |
| **What is your compliance level with the following measures:** | | | | | | | | | | | | | | | | | | |  |
|  | | | | *Never* | | | *Rarely* | | | | *Frequently* | | | | *Always* | | *No answer* | | |
| Mandatory use of mask | | | | 0.1 | | | 1.5 | | | | 16.0 | | | | 82.0 | | 0.4 | | |
| Social distancing (1,5 meters) | | | | 0.3 | | | 8.1 | | | | 43.1 | | | | 48.1 | | 0.4 | | |
| Stay home, if possible | | | | 1.1 | | | 6.7 | | | | 40.0 | | | | 51.9 | | 0.3 | | |
| **Indicate your age:** | | | | | | | | | | | | | | | | | | |  |
| Less than 18 years | | | | | | | | | | | | | | | | | | | 0.9 |
| Between 19 and 25 years | | | | | | | | | | | | | | | | | | | 30.6 |
| Between 26 and 32 years | | | | | | | | | | | | | | | | | | | 20.9 |
| Between 33 and 45 years | | | | | | | | | | | | | | | | | | | 27.4 |
| Between 46 and 64 years | | | | | | | | | | | | | | | | | | | 18.4 |
| Between 65 and 79 years | | | | | | | | | | | | | | | | | | | 1.9 |
| More than 80 years | | | | | | | | | | | | | | | | | | | 0.1 |
| **What is your gender identification?** | | | | | | | | | | | | | | | | | | |  |
| *Male* | | | | | *Female* | | | | | | | | | *Other* | | | | | |
| 37.9 | | | | | 61.7 | | | | | | | | | 0.4 | | | | | |
| **What is your state´s residence?** | | | | | | | | | | | | | | | | | | |  |
| *AC* | 0.1 | *AL* | | | | 0.1 | | | *AM* | | | 1.4 | | | | *AP* | | 0.0 | |
| *BA* | 1.5 | *CE* | | | | 0.6 | | | *DF* | | | 2.1 | | | | *ES* | | 0.2 | |
| *GO* | 2.4 | *MA* | | | | 0.1 | | | *MG* | | | 7.3 | | | | *MS* | | 1.8 | |
| *MT* | 0.3 | *PA* | | | | 0.1 | | | *PB* | | | 0.4 | | | | *PE* | | 0.4 | |
| *PI* | 0.1 | *PR* | | | | 5.9 | | | *RJ* | | | 5.3 | | | | *RN* | | 0.4 | |
| *RO* | 0.0 | *RR* | | | | 0.0 | | | *RS* | | | 1.1 | | | | *SC* | | 1.1 | |
| *SE* | 0.2 | *SP* | | | | 67.0 | | | *TO* | | | 0.1 | | | |  | |  | |
| **What is your highest education level achieved until the date?** | | | | | | | | | | | | | | | | | | |  |
| Elementary School | | | | | | | | | | | | | | | | | | | 0.5 |
| High School | | | | | | | | | | | | | | | | | | | 14.4 |
| University – Degree | | | | | | | | | | | | | | | | | | | 40.5 |
| University – MBAs and Specializations | | | | | | | | | | | | | | | | | | | 20.8 |
| University – Master | | | | | | | | | | | | | | | | | | | 14.1 |
| University - Doctorate | | | | | | | | | | | | | | | | | | | 9.7 |
| **In 1 to 7 scale, choose the number which matches with your political positions:** | | | | | | | | | | | | | | | | | | |  |
| *1 – Far Left* | *2* | *3* | | | | *4 - Center* | | | *5* | | | *6* | | | | *7 - Right* | | *No answer* | |
| 2.3 | 14.3 | 19.7 | | | | 21.6 | | | 11.7 | | | 7.7 | | | | 2.7 | | 20.0 | |
