# Supplementary material for: Perceptions of institutional performance and compliance to non-pharmaceutical interventions: How performance perceptions and policy compliance affect public health in a decentralized health system
Source: PLoS One. 2023 May 12;18(5):e0285289. doi: 10.1371/journal.pone.0285289 (PMC10180683; doi:10.1371/journal.pone.0285289)
Supplement: Attachment — Submitted filename: Response to Reviewers.docx [file pone.0285289.s005.docx]

**PONE-D-22-28667R1**

**Perceptions of institutional performance and compliance to non-pharmaceutical interventions: how performance perceptions and policy compliance affect public health in a decentralized health system**

Editor’s/Reviewers’ requirements

1) SUS is not fragmented but decentralized. So it was clear the effort that was made to adjust the text to this issue; however, it still missed some indications of the SUS as a fragmented system, which is quite a mistaken assessment, since there is a huge effort of federative coordination within the SUS structure, which is the largest existing public health system.

a. Some indications need to be corrected on the title, pages 3 (abstract) and 16 (conclusions)

*Revision:* Dear Editor and Reviewer, thank you very much for the contribution. Following your advice, we changed all the terms (4) “fragmented” to *“decentralized”* in the text (title, abstract and 2 in the conclusions).

2) Brazil is not a low-middle income country but an Upper Middle Income Country, as can be seen on the World Bank's website (https://data.worldbank.org/?locations=BR-XT)

*Revision:* Dear Editor and Reviewer, thank you very much for the contribution. Following your advice, we changed the term “low middle-income country” to *“upper middle-income country”* in the text (page 5).

3) It is important to make it clear to the reader that the sample is convenient and not representative. Thus, at the end of the introduction, I ask you to insert the word "convenience" in the following sentence.

For this purpose, we rely on an online "convenience" national survey to explore (...)

*Revision:* Dear Editor and Reviewer, thank you very much for the contribution. Following your advice, we changed the sentence to *“For this purpose, we rely on an online convenient national survey to explore (…)”.*

Journal requirements

1) Please review your reference list to ensure that it is complete and correct. If you have cited papers that have been retracted, please include the rationale for doing so in the manuscript text, or remove these references and replace them with relevant current references. Any changes to the reference list should be mentioned in the rebuttal letter that accompanies your revised manuscript. If you need to cite a retracted article, indicate the article’s retracted status in the References list and also include a citation and full reference for the retraction notice.

*Revision:* Dear journal, we revised our reference list and citations, and it was correct, so no changes were made.
